# Lake Superior evaporation: A long-term eddy covariance dataset at Stannard Rock Lighthouse (2008–2022)

**DOI:** 10.1038/s41597-024-03940-7

**Published:** 2024-10-18

**Authors:** Erin M. Nicholls, Christopher Spence, Newell Hedstrom, John D. Lenters, Peter D. Blanken

**Affiliations:** 1 Environment and Climate Change Canada, Saskatoon, SK Canada; 2 University of Michigan Biological Station, Pellston, MI USA; 3 Department of Geography, University of Colorado, Boulder, CO USA

**Keywords:** Hydrology, Hydrology, Atmospheric dynamics

## Abstract

Robust, accurate, and direct measurements of evaporation and related energy fluxes on the Laurentian Great Lakes are necessary to understand the large historical range in water levels, regional climatology, lake hydrodynamics, and lake-effect snowfall, all of which inform water management. Despite the societal and scientific importance of this information, few long-term, full-year, *in situ* measurements exist due to logistical, financial, and safety-related challenges. We present 15 years (2008–2022) of eddy covariance data from Stannard Rock, a historic lighthouse on Lake Superior located 38 km southeast of Manitou Island and 72 km north of Marquette, Michigan. We provide information about the site and instrumentation, as well as data availability and processing. Analysis of this unique long-term dataset, available through the AmeriFlux network (US-GL1), will improve our ability to understand the drivers and patterns of large-lake surface energy fluxes and will advance predictions of evaporative regimes over Lake Superior.

## Background & Summary

The Laurentian Great Lakes are the world’s largest inland freshwater ecosystem, with a total surface area greater than 2.4 × 10^5 ^km^2^. The Great Lakes drainage basin is home to over 38 million people^1^ and serves as a critical resource for both the United States and Canada through power production, shipping, industry, agriculture, and recreation. The lakes play an important role in controlling local and regional climatological and ecological interactions through complex biophysical and biogeochemical processes^2^. Lake Superior is the largest freshwater lake in the world with respect to surface area (82.1 × 10^3 ^km^2^). Due to its large volume (12.1 × 10^3^ km^3^), the heat capacity is substantial, thus changes in the lake’s energy content have considerable impacts on the regional climate^3,4^. Regional climate simulations of local terrestrial air temperature, evaporation, and precipitation are significantly improved when over-lake processes are included^5^.

While estimates of lake evaporation rates are essential to quantify the regional water balance and climate dynamics, and are crucial for predicting changes in water level, direct measurements of over-lake evaporation are scarce, especially for large lakes. In absence of direct measurements over the Great Lakes, early estimates of surface energy fluxes (including evaporation, expressed as the latent heat flux) were modelled using turbulent transfer equations and shore-based meteorological data adjusted for over-lake conditions^6^. It is well known that the meteorological conditions occurring offshore over large lakes can differ significantly from those measured onshore^7^. Thus, model-based estimates of evaporation will be inaccurate unless provisions are made to account for such differences or, preferably, if direct evaporation measurements are made over the lake itself. The turbulent fluxes of scalars (e.g., heat, water vapor, momentum) can be directly measured using the eddy covariance or other micrometeorological techniques, provided that a stable platform is used to mount instruments and facilitate maintenance. This is clearly a barrier to measurements over large lakes, especially in the fall and winter seasons when lake-air surface exchanges are large^8,9^, dynamic lake ice can be present^10^, and winter limnological processes remain a key knowledge gap^11^. In recent decades, flux networks (i.e., AmeriFlux, FLUXNET, etc.) have expanded to include flux measurements over a wide range of terrestrial ecosystems^12^, yet measurements over lakes continue to be rare. For example, not including Stannard Rock, currently only 9 out of 635 sites within the AmeriFlux network are classified as ‘Water Bodies’ and notably, all represent small, freshwater lakes.

To address the absence of direct, over-lake evaporation measurements over the Great Lakes, and Lake Superior in particular, Stannard Rock Lighthouse was instrumented with eddy covariance instrumentation in June 2008 and has since operated nearly continuously^8,9^. This effort was initially coordinated by the International Upper Great Lakes Study (IUGLS) through the International Joint Commission (IJC)^13^ and by 2013 grew to a total of five eddy covariance sites on the Great Lakes, thereby forming the Great Lakes Evaporation Network (GLEN; https://superiorwatersheds.org/GLEN/)^14^. The GLEN data have been used to improve both Canadian and American operational atmospheric forecast models^15–17^ including those predicting lake-effect snowfall^18,19^. These studies used short-term data to calibrate and validate models and/or compare with additional observational datasets. Here, we present the entire 15-year collection (2008–2022) of eddy covariance and meteorological data from Stannard Rock Lighthouse, encompassing a wide range of lake and meteorological conditions from the longest-running GLEN site on the Great Lakes. The data are available through https://ameriflux.lbl.gov/sites/siteinfo/US-GL1. We describe the site location and history, instrumentation, data availability, seasonal and interannual variability, and associated data processing and quality control methods.

## Methods

### Site description

**Fig. 1:**
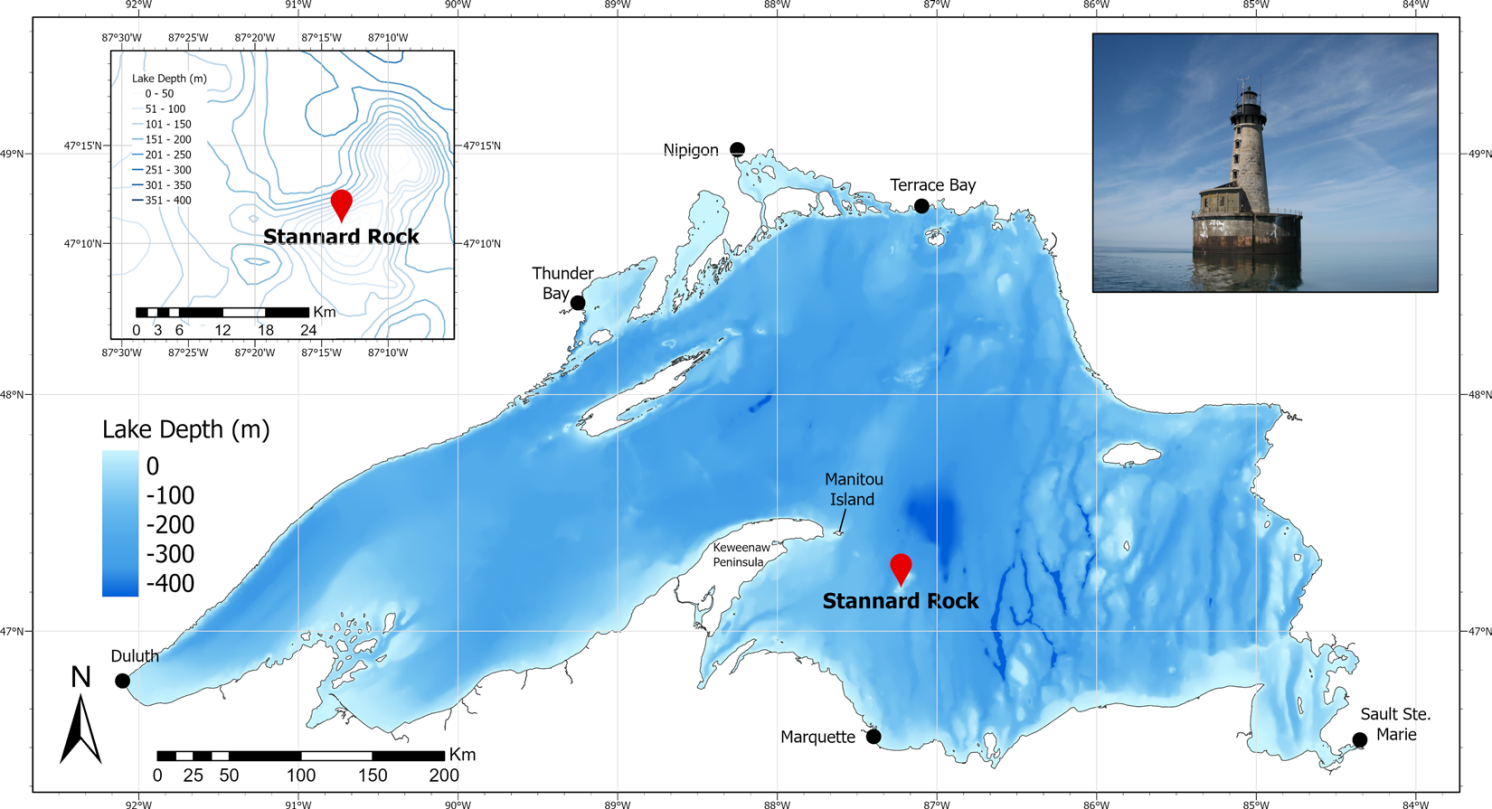
Bathymetric map of Lake Superior including the location of Stannard Rock Lighthouse (red marker). A photo of the lighthouse in mid-summer on a calm day is shown in the upper right. Contours of water depth surrounding Stannard Rock are shown in the upper left inset. This spatial data was supplied by NOAA National Geophysical Data Center’s Marine Geology & Geophysics Division (NGDC/MGG) and the NOAA Great Lakes Environmental Research Laboratory (GLERL).

The size, northern location, and volatile weather conditions make Lake Superior a challenging location for meteorological observations. As such, Stannard Rock Lighthouse provides an ideal location for measuring year-round, over-lake water measurements due to its offshore location, height and stability, and the absence of any nearby land surface (Fig. 1). The limestone cylindrical historic lighthouse (47.183°N, 87.225°W) was completed in 1882 and stands roughly 38 m above the water surface on the edge of a shallow shoal 38 km from the nearest shore (the Keweenaw Peninsula in Michigan). Since 1984, Stannard Rock has been reporting meteorological data as a National Oceanic and Atmospheric Administration (NOAA) National Data Buoy Center C-MAN station (STDM4). While the water is relatively shallow around the reef (~3–5 m deep), depths in the surrounding source area for flux measurements (~6 km upwind during the active evaporation season) range from 150–300 m^8^.

### Instrumentation

Meteorological and eddy covariance instrumentation were installed on a mast fastened to the top of the lighthouse 39.2 m above the mean water surface (Table 1). The eddy covariance and supporting meteorological measurements reported here were made between June 1, 2008 and December 31, 2022 with short periods of missing data as reported below due to power failures and data quality control. Turbulent fluxes of sensible (H) and latent (LE) heat and carbon dioxide (CO_2_) were calculated from 10-Hz measurements of vertical wind speed (w), temperature, water vapor density and carbon dioxide. Wind speed was measured using a 3-D sonic anemometer (model CSAT-3, Campbell Scientific, Logan, UT) (Table 1). Water vapor and carbon dioxide densities were measured using both an open path CO_2_/H_2_O gas analyzer (model LI-7500, LI-COR Biosciences, Lincoln, NE) located 15 cm away from the CSAT-3 and an additional measurement of LE was made with an open path krypton hygrometer (model KH2O, Campbell Scientific, Logan, UT) (Table 1). Means and covariances of the high-frequency sampled data were collected at 30-minute intervals using a datalogger (model CR3000, Campbell Scientific, Logan, UT). Power was supplied by eight 12-V deep cycle marine batteries charged by six 80-W solar panels. Data were stored on-site and either downloaded during site visits or transmitted via radio telemetry to a shore-based station at Big Bay, Michigan (between June 2008 and August 2014) and via NOAA’s Great Lakes Environmental Research Laboratory’s Realtime Coastal Observation Network (after August 2014). The data were originally recorded in Eastern Daylight Time (EDT/−4:00 UTC) from the initial installation until March 17, 2015 at 20:30, when the logger began recording in UTC. As per AmeriFlux standards, all data reported in this dataset was shifted to account for these time zone changes and reported as local standard time (−5:00 UTC).

**Table 1:** Meteorological and eddy covariance data variables available through AmeriFlux (US-GL1).

Variable	AmeriFlux Variable Name	Sensor(s)	Sensor Height (m above mean water level)	Units	Processing
**Latent heat flux**	LE_1_1_A	CSAT-3/LI-7500 and KH20	39.2	W m^−2^	See Data processing and quality control
	LE_1_1_1	CSAT-3/LI-7500	39.2	W m^−2^	See Data processing and quality control
	LE_1_1_2	CSAT-3/KH20	39.2	W m^−2^	See Data processing and quality control
**Sensible heat flux**	H_1_1_1	CSAT-3/LI-7500	39.2	W m^−2^	See Data processing and quality control
**Atmospheric pressure**	PA	LI-7500	39.2	kPa	Filtered below 94 kPa
**Relative humidity**	RH	HMP-45C	39.2	%	Calculated (TA and e_a_)
**Incoming shortwave radiation**	SW_IN	SPLite pyranometer	39.2	W m^−2^	Filtered above 1100 and below 0 W m^−2^
**Incoming longwave radiation**	LW_IN	CGR4 pyrgeometer	39.2	W m^−2^	N/A
**Friction velocity**	USTAR	CSAT-3	39.2	m s^−1^	N/A
**Horizontal wind speed**	WS	RM Young Anemometer	39.2	m s^−1^	N/A
**Air temperature**	TA	HMP45C	39.2	°C	N/A
**Vapour pressure deficit**	VPD	HMP45C	39.2	hPa	Calculated (TA and e_a_)
**Momentum flux**	TAU	CSAT-3	39.2	kg m^−1^ s^−2^	N/A
**Wind direction**	WD	RM Young Anemometer	39.2	Degrees (°)	N/A
**Carbon dioxide flux**	FC	CSAT-3/LI-7500	39.2	µmolCO_2_ m^−2^ s^−1^	See Data processing and quality control
**Carbon dioxide density**	CO2	LI-7500	39.2	umolCO_2_ mol^−1^	See Data processing and quality control
**Water temperature**	TW	Apogee- IRR-P/SI-111	33.3	°C	Estimated – see Data processing and quality control

Ancillary meteorological variables were measured at a 5-s sampling rate and averaged over 30 minutes. Air temperature (TA; °C) and relative humidity (RH; %) were measured with a shielded probe (model HMP45C, Vaisala, Helsinki). Atmospheric pressure (PA; kPa) was measured using a pressure transducer located inside the LI-7500 control box. A vane anemometer (model 05106, RM Young, Traverse City, MI) measured horizontal wind speed (WS; m s^−1^) and direction with respect to true north (WD, °). Incoming shortwave radiation (SW_IN) was measured using a pyranometer (SPLite, Kipp & Zonen, Delft, Netherlands) and incoming longwave radiation (LW_IN) was measured with a pyrgeometer (CGR4, Kipp & Zonen, Delft, Netherlands).

Water surface temperature (TW) was estimated using an infrared thermometer (IRT); the IRR-P model was used until September 2017 when this was swapped with a model SI-111 (Apogee, Logan, UT) (Table 1). While TW is included in the dataset (TW), it is advised to use this variable with caution; this measurement was unreliable at times (primarily the winter months) due to condensation or frost formation inside the instrument cavity, as well as interference from other surfaces (lighthouse or sky). Surface water temperature data can also be obtained from the Great Lakes Surface Environmental Analysis (GLSEA) (http://coastwatch.glerl.noaa.gov) (as used by Spence *et al*.^9^), which represents the daily water surface temperature during the cloud-free periods of the previous day’s satellite imagery at the pixel closest to Stannard Rock.

### Data processing and quality control

Estimations of gas fluxes using the eddy covariance method require simultaneous high frequency measurements of vertical wind speed and gas densities. Because much of the turbulent transport occurs at frequencies between 0.0001 and 5 Hz, a sampling frequency of 10–20 Hz is recommended for eddy covariance measurements^20^. Uniform terminology and methodology have been developed and standardized to post-process eddy covariance measurements, largely due to efforts by networks such as FluxNet, AmeriFlux, AsiaFlux, ICOS, NEON, etc. In recent decades, it has become standard practice for eddy covariance towers to collect and store these large datasets of raw high frequency data for subsequent processing (i.e., unit conversion, despiking, applying calibrations, coordinate rotation, accounting for time delays, detrending, and averaging) and corrections (i.e., frequency response, density fluctuations, flux storage, etc.)^20^. Recently, the technology has evolved to allow for processing and correction of the raw fluxes on-site to provided fully processed fluxes in near real-time (e.g., SmartFlux). This technology was not available when Stannard Rock was first instrumented. Limited site access (open water season only) together with the limited on-site data storage capacity dictated that only the 30-minute statistics required for turbulent flux calculations could be retained. Traditional dataloggers do not have the computational capacity to perform full processing on the high frequency data but can store the data for limited time periods and calculate and store half-hour fluxes. Uncorrected half-hour fluxes were computed directly on the logger using a 30-minute block averaging period.

The half-hour flux measurements downloaded from the datalogger were post-processed with the following filters and corrections. Wind speeds in the x, y, and z direction are represented by u, v, and w respectively. Latent and sensible heat and carbon dioxide fluxes were corrected with 2-D coordinate rotation. The first rotation is about the z-axis (vertical) and aligns the u- and v- wind directions on the x-y plane, and the second rotation is about the x direction and aligns the w wind into the z direction^21,22^. Although the primary objective of data collection at Stannard Rock was to quantify the evaporative flux, additional preliminary measurements of the carbon dioxide concentration and flux from the LI-7500 are also included in this dataset. Carbon data reported in this dataset includes turbulent fluxes of CO_2_ with no storage correction (FC, µmol m^−2^ s^−1^) and CO_2_ density in mole fraction of wet air (CO2), which was originally output on the datalogger as average CO_2_ density (mg m^−2^ s^−1^) and converted into µmol mol^−1^ using air temperature and pressure in post-processing. Webb, Pearman, and Leuning terms were applied to account for density fluctuations for water vapor and CO_2_^23^. Sonic path length, high-frequency attenuation and sensor separation were accounted for according to Horst^24^ and Massman^25^, and the oxygen absorption correction for the KH2O hygrometer was also applied (see Technical Validation). Latent and sensible heat fluxes were assumed to be unrealistic above an absolute value of 1000 W m^−2^ and were removed. Over the 15 year period, these thresholds resulted in an overall removal of 0.25% (sensible heat), 0.13% (KH20 latent heat), and 1.4% (LI7500 latent heat). Both carbon flux (FC) and carbon dioxide mole fraction in wet air (CO2) were assumed to be unrealistic above 1000 µmol m^−2^ s^−1^ and 1000 µmol mol^−1^ respectively. Spikes in latent and sensible heat and carbon fluxes and densities (often due to periods of precipitation) were identified by computing the mean and standard deviation over a moving, overlapping window of 336 half-hours (7 days), similar to Shao *et al*.^26^, and were removed when the flux was more than 1.5 standard deviations from the moving window’s mean. While Vickers and Mahrt^27^ use a threshold of 3.5 standard deviations from the mean, a conservative value of 1.5 was chosen due to the noisy nature of over-lake data at this particular site^27^. This process was repeated twice for latent and sensible heat, as well as for carbon dioxide fluxes and densities. The despiking resulted in further removal of 12.8, 9.4, and 11.8% of the sensible and latent (KH20 and LI7500) heat fluxes respectively. No detrending was performed. As per AmeriFlux standards, no friction velocity (USTAR, m s^−1^) filtering was performed.

**Fig. 2:**
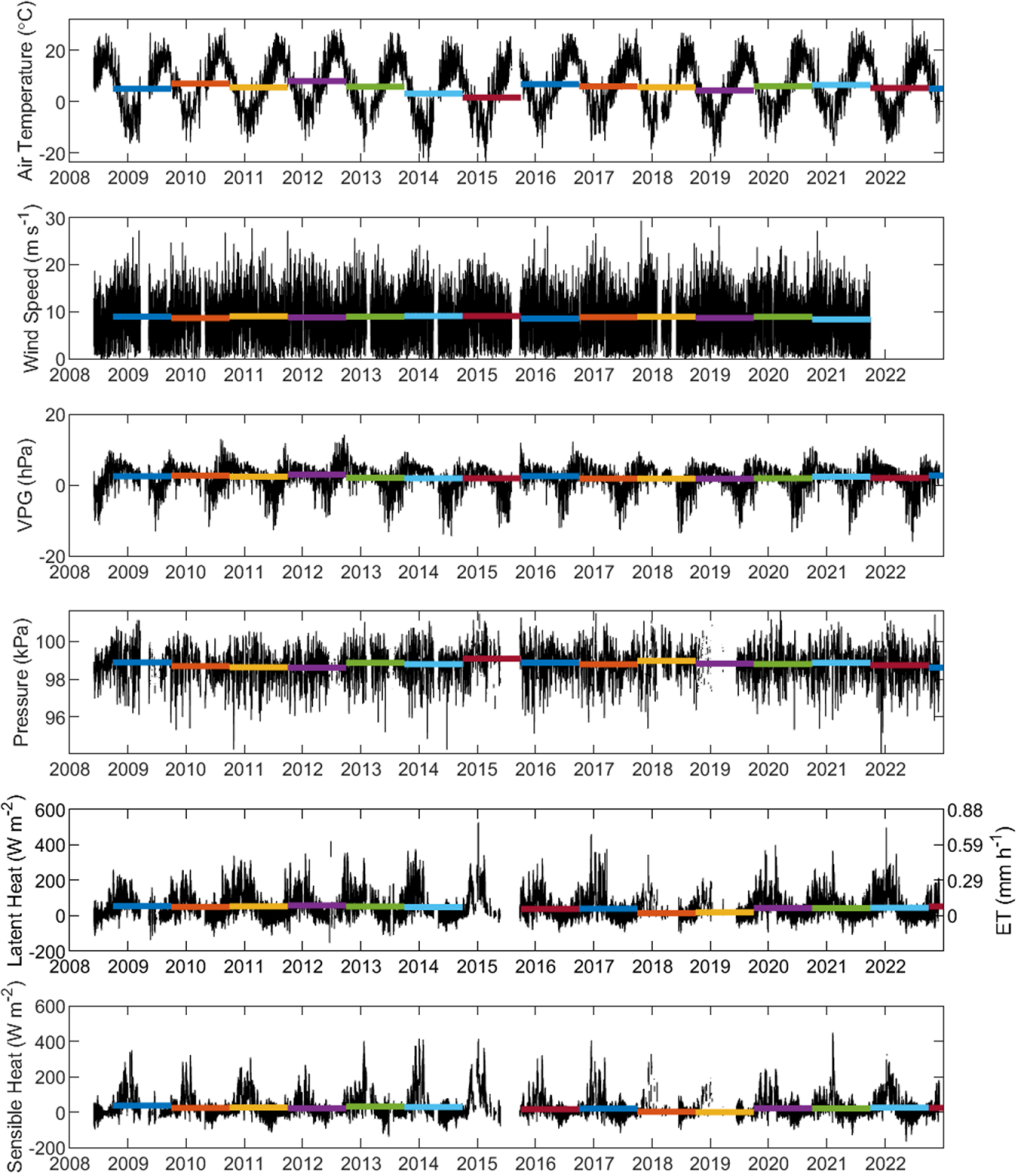
Half hour measurements of air temperature, wind speed, vapor pressure gradient (VPG), barometric pressure, and aggregated latent and sensible heat fluxes (black). Colored bars denote the annual mean of each variable using ungapfilled data within each water year (1 Oct–30 Sept).

Quality control procedures for the accompanying meteorological data included filtering out data values beyond the physical range expected or possible for each variable (Table 1). Values of relative humidity were removed above and below 100 and 0%, respectively. Wind speed data from the RM Young were not available after Oct 13, 2021, due to instrument malfunction. Incoming shortwave radiation data were removed below 0 and above 1100 W m^−2^. Vapor pressure deficit (VPD) within the dataset was calculated using relative humidity and air temperature at the sensor height. Notably, this is different than the vapor pressure gradient (VPG), which can be calculated as the saturated vapor pressure at the water surface (calculated using GLSEA surface water estimates) minus the saturated vapour pressure at the measurement height. The final half-hour time series of select AmeriFlux data variables following these post-processing procedures are shown in Fig. 2.

## Data Records

The dataset is available for download at https://ameriflux.lbl.gov/sites/siteinfo/US-GL1, with this section being the primary source of information on the availability and content of the data being described. Variables within the AmeriFlux network are comprised of a base name, which indicates the measured or derived physical quantity, and a qualifier that is appended as a suffix to the base name. Within the US-GL1 dataset, there are three versions of the latent heat flux (LE_1_1_A, LE_1_1_1, LE_1_1_2). The suffixes represent _H_V_R, as the horizonal (H) and vertical (V) plane of measurement, followed by the replicate number (R). The qualifier “_A” in the replicate location denotes aggregated data, where the output from the IRGA was used first and gap-filled with krypton hygrometer data (Table 1). The variable LE_1_1_1 represents latent heat computed using the LI-7500, and LE_1_1_2 is latent heat from the KH20 as they are both in the same horizontal and vertical planes and serve as replicates. All turbulent fluxes and meteorological data are averaged over half-hour intervals, indicated by the TIMESTAMP_START and TIMESTAMP_END variables and formatted numerically as YYYYDDMMHHMM (year, day, month, hour, minute).

**Fig. 3:**
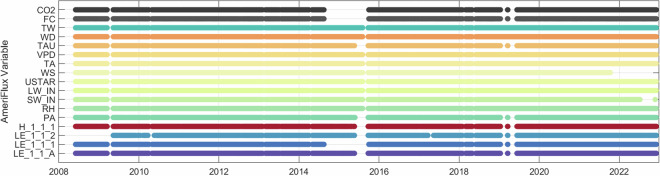
Data availability throughout the 2008–2022 study period for each AmeriFlux variable. Note that only long periods of missing data are visible here due to the length of data shown.

While the dataset is relatively continuous, periods of missing data occurred, primarily due to insufficient power, with notably long periods from March 20-May 5, 2009, May 25-Sept 23, 2015, May 1-June 1, 2018, and Dec 16, 2018-June 12, 2019 (Fig. 3). These power failures resulted in large periods of missing raw turbulent flux or meteorological data, particularly during water years 2014–2015, 2017–2018, and 2018–2019 (Table 2). Once aggregated and filtered according to methods described above, latent heat (LE_1_1_A) was missing for 36% of the timeframe presented in this dataset (Figs. 2, 3; Table 2). This is comparable to the latent heat measured with the LI-7500 (LE_1_1_1) and KH20 (LE_1_1_2) which were missing 41% and 53% of the time, respectively. In the 2014–2015 water year, the LI-7500 was not functioning and therefore LE_1_1_1 was missing for the entire period (Table 2) and the resulting aggregated latent heat data was largely missing (72%). The meteorological variables had comparably fewer missing data overall, particularly the incoming shortwave and longwave radiation which, on average, were both missing less than 8% of the dataset. As per AmeriFlux standards, any missing data were replaced with −9999 in the dataset.

**Table 2:** Percent (%) missing for dataset variables for each water year (Oct 1 – Sept 30) and average for all years.

Water Year	LE_1_1_A	LE_1_1_1		LE_1_1_2		H_1_1_1		PA	TA	SW_IN	LW_IN	USTAR	WS	FC		CO2	
	Final	Raw	Final	Raw	Final	Raw	Final	Final	Final	Final	Final	Final	Final	Raw	Final	Raw	Final
2008–2009	48.3	37.5	48.9	67.7	84.2	37.5	48.2	36.9	14.4	14.4	14.4	15.0	14.4	37.5	51.9	36.9	53.9
2009–2010	24.2	9.0	24.7	69.4	75.0	9.0	24.5	8.9	7.8	7.8	7.8	7.9	7.8	9.0	28.4	8.9	30.7
2010–2011	20.5	5.3	22.8	10.6	30.2	5.3	22.0	4.6	0.4	0.4	0.4	1.1	0.4	5.3	25.9	4.6	28.4
2011–2012	30.9	18.7	32.4	12.4	39.8	18.7	33.8	18.1	2.6	2.6	2.6	3.2	2.6	18.9	35.2	18.3	37.2
2012–2013	33.1	19.5	35.0	35.4	54.0	19.4	33.1	18.7	6.4	6.4	6.4	7.7	6.4	19.8	37.3	18.7	38.7
2013–2014	29.0	13.4	39.1	28.3	44.7	13.4	29.8	12.5	7.7	7.7	7.7	9.0	7.7	13.5	41.9	12.4	44.4
2014–2015	72.1	62.5	99.7	32.3	72.1	62.4	68.9	62.3	18.5	18.5	18.5	19.3	18.5	62.5	99.7	62.3	99.7
2015–2016	17.9	4.4	21.2	7.9	26.8	4.4	21.2	4.3	0.0	0.0	0.0	0.1	0.0	4.4	24.0	4.3	27.3
2016–2017	25.6	12.2	27.8	39.4	53.1	12.2	27.6	7.9	2.1	2.1	2.1	6.4	2.1	12.2	30.8	7.9	32.9
2017–2018	71.1	65.3	71.7	68.6	74.4	65.2	71.7	28.9	16.2	16.2	16.2	65.2	16.2	65.3	72.9	28.9	74.0
2018–2019	71.0	65.7	71.6	9.7	73.7	65.7	71.4	65.4	0.0	0.0	0.0	0.9	0.0	65.8	72.3	65.4	73.5
2019–2020	22.4	9.6	25.5	15.7	34.6	9.6	25.7	8.8	4.2	4.3	4.3	5.1	4.3	9.7	29.6	8.8	30.9
2020–2021	18.9	5.5	21.5	18.6	35.9	5.5	21.5	4.9	0.1	0.1	0.1	0.8	0.1	5.6	24.4	4.9	27.2
2021–2022	22.5	10.9	27.6	18.4	39.8	10.9	26.8	9.6	1.0	23.0	1.0	2.4	96.3	10.9	30.7	9.5	32.3
Average	36.3	24.2	40.7	31.0	52.8	24.2	37.6	20.8	5.8	7.4	5.8	10.3	12.6	24.3	43.2	20.8	45.1

Raw indicates available data downloaded from the datalogger. Final indicates the final value reported in the AmeriFlux dataset after being processed as per Data processing and quality control (if applicable).

## Technical Validation

An important part of maintaining high data quality is the regular maintenance and substitution of sensors with newly calibrated units. The krypton hygrometer at Stannard Rock was installed in May 2009 (SN1229) and replaced in June 2010 (1228), September 2015 (SN1229), and May 2017 (SN1662) (Table 3). The hygrometer was cleaned at each site visit (roughly twice per year). If the signal strength fell within the calibration specifications, the instrument was left running. If the signal strength was too low and cleaning did not improve the signal or the sensor failed, the hygrometer was replaced and re-calibrated. Calibrations of the hygrometer were performed by Campbell Scientific. After each calibration, a new value of xkw (the path length times the absorption coefficient for water vapour) was written directly into the logger program and used to calculate LE. The output values of xkw and the dates of each calibration are summarized in Table 3. The LI-7500 was replaced in July 2009, May 2012, and September 2015.

**Table 3:** Instrument serial numbers and calibration values for KH20.

KH20		
Dates (dd/mm/yyyy)	Serial number	xkw
08/05/2009 to 26/05/2010	1229	−0.188
26/05/2010 to 04/09/2015	1228	−0.209
04/09/2015 to 11/05/2017	1229	−0.197
11/05/2017 to 31/12/2023	1662	−0.173

**Fig. 4:**
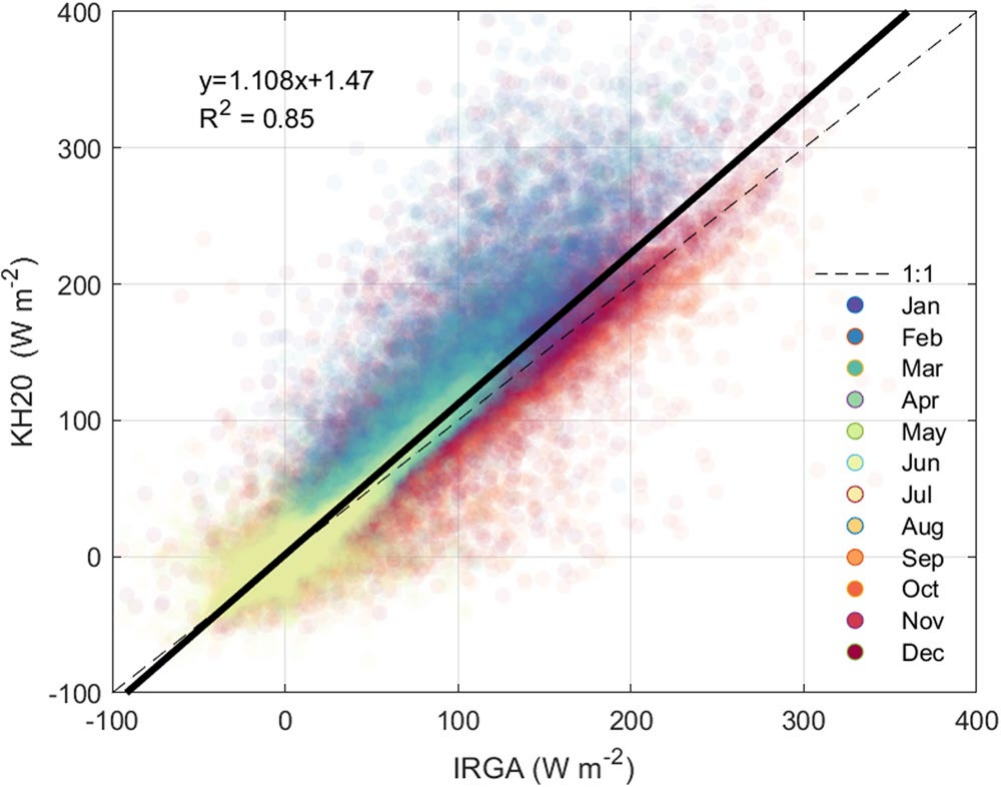
The relationship between half hour latent heat flux using the LI7500 IRGA and KH20 krypton hygrometer. Colors represent month of measurement. The solid line represents the linear regression over the entire study period. The dashed line represents the 1:1 line.

As discussed above, eddy covariance measurements, particularly in this environment, have associated uncertainties. Energy balance closure (EBC), calculated as the statistical regression of turbulent energy fluxes against available energy (net radiation less stored energy), is often used to evaluate eddy covariance measurements^28,29^. Over land, most studies over the last few decades have shown turbulent fluxes are underestimated, with a mean imbalance of 20%, either due to underestimation of turbulent fluxes, or overestimation of available energy^29,30^. This discrepancy has been attributed to sampling errors associated with different measurement source areas, systematic bias in instrumentation, energy sinks that are unaccounted for, loss of low or high frequency contributions to the flux, and/or neglected advection of scalars. Evaluating EBC at Stannard Rock is not feasible due to the large heat storage component of the lake itself and the large uncertainty of this term. Other evaluations of eddy covariance performance and uncertainties have used multiple tower experiments^31–34^, although these are also challenging when measuring fluxes above a remote lake location. We do, however, have two measurements of latent heat flux that allow for comparison of the half hour latent fluxes (Fig. 4). Comparison of the LI-7500 and KH20 indicate close agreement between the estimates derived by the two sensors. Overall, the LI-7500 and KH20 are within 11% of each other, with the latter producing slightly higher latent heat flux estimates. The discrepancy between the two instruments falls within the natural range of variance of eddy covariance measurements. For example, the typical error for latent heat even over terrestrial environments can be ~5–20%^35^.

There is a seasonal pattern to differences between the two sensors, with the LI-7500 tending to provide estimates lower than those from the KH20 from December until April (Fig. 2). Latent heat is near-zero or negative in the summer months, which can occur during periods of fog and condensation when the vapor pressure in the warmer atmosphere exceeds the cold lake surface. Uncertainty between the sensors is highest in these summer months when turbulent fluxes were low (R^2^ = 0.59, 0.42, and 0.42 and slopes of 0.75, 0.49, and 0.54 in May, June, and July respectively). The best fit between the two sensors (i.e., R^2^ > 0.76) occurs when fluxes are their highest from August to April. It should be noted that these relationships were developed using the post-processed data available through AmeriFlux, but could likely be improved using further cleaning techniques not applied here (i.e. friction velocity thresholds).

## Usage Notes

While every effort has been made to ensure the highest quality data are reported here, the harsh and challenging conditions of measuring evaporative fluxes above a large, cold, deep lake such as Lake Superior should be kept in mind when using this dataset. As discussed above, the high frequency measurements that are often recorded and processed at other eddy covariance sites were not available due to the Stannard Rock’s remote location and older technology (i.e., starting in 2008). The resulting dataset presented here provide a useful and unique estimation of evaporative fluxes over a long record that can be used to further understand variations in water levels, regional climatology, lake hydrodynamics, and lake-effect snowfall, as well as to inform water management. Over-water eddy covariance measurements are inherently challenging. For example, it is possible that the instrument height on the lighthouse was occasionally (e.g., during very atmospherically stable conditions in spring and early summer) above the temperature inversion layer, and this is reflected in higher uncertainty during these periods (Fig. 4). Users are advised to be particularly cautious when using flux measurements obtained when atmospheric conditions are highly stable during summer. Additionally, over terrestrial environments, diurnal patterns are more easily discernable, and evaporative fluxes are typically less noisy. Therefore, within our spike removal decisions, it is possible that some real data were filtered out.

Given that some of the above uses require seasonal totals of evaporation from the lakes, we advise the user to be aware of these limitations. Spence *et al*.^9^ showed that the transient nature of synoptic scale air masses can result in brief and intense evaporation events. It is important that if the user of this dataset were to gap-fill missing data, they keep in mind that short events can dominate the seasonal and monthly evaporation volume (i.e., up to half the evaporation occurring 20% of the time)^9^. As outlined within the Data Records section, certain periods of measurement may be more complete than others and therefore the treatment of data interpretation is dependent on the research objective and period of interest of the specific user. Despite the considerations of missing data periods and filtered values, this dataset is widely applicable to compare shore-based meteorological measurements to over-lake conditions, and assess seasonal dynamics, interannual variability, and the impact of teleconnections on large-lake evaporation. Furthermore, various gap-filling techniques are available (including the use of models and machine learning), and such methods could be applied to assist in calculating annual total evaporation.The data presented here represent a 15-year effort to measure surface atmospheric and energy balance conditions over Lake Superior as part of the Great Lakes Evaporation Network (and now AmeriFlux). These data are the result of the longest and most complete energy flux measurement campaign over any of the Laurentian Great Lakes. They provide insight into hydrometeorological processes that occur over large temperate water bodies that are important for assessing lake response to climate variability and change. Operational environmental prediction systems in both Canada and the United States have been improved because of these data. The purpose of making the data available is to continue to support such activities and support research and management of climate and water in the Laurentian Great Lakes and elsewhere.

## Data Availability

This dataset was compiled using MATLAB (Version R2022b), however no custom code is required for use of this dataset.
